# Potential Mechanisms for Why Not All Antipsychotics Are Able to Occupy Dopamine D_3_ Receptors in the Brain *in vivo*

**DOI:** 10.3389/fpsyt.2022.785592

**Published:** 2022-03-24

**Authors:** Béla Kiss, Balázs Krámos, István Laszlovszky

**Affiliations:** ^1^Pharmacological and Drug Safety Research, Gedeon Richter Plc., Budapest, Hungary; ^2^Spectroscopic Research Department, Gedeon Richter Plc., Budapest, Hungary; ^3^Global Medical Division, Gedeon Richter Plc., Budapest, Hungary

**Keywords:** schizophrenia, antipsychotics, D_3_ receptor, D_2_ receptor, dopamine, brain occupancy

## Abstract

Dysfunctions of the dopaminergic system are believed to play a major role in the core symptoms of schizophrenia such as positive, negative, and cognitive symptoms. The first line of treatment of schizophrenia are antipsychotics, a class of medications that targets several neurotransmitter receptors in the brain, including dopaminergic, serotonergic, adrenergic and/or muscarinic receptors, depending on the given agent. Although the currently used antipsychotics display *in vitro* activity at several receptors, majority of them share the common property of having high/moderate *in vitro* affinity for dopamine D_2_ receptors (D_2_Rs) and D_3_ receptors (D_3_Rs). In terms of mode of action, these antipsychotics are either antagonist or partial agonist at the above-mentioned receptors. Although D_2_Rs and D_3_Rs possess high degree of homology in their molecular structure, have common signaling pathways and similar *in vitro* pharmacology, they have different *in vivo* pharmacology and therefore behavioral roles. The aim of this review, with summarizing preclinical and clinical evidence is to demonstrate that while currently used antipsychotics display substantial *in vitro* affinity for both D_3_Rs and D_2_Rs, only very few can significantly occupy D_3_Rs *in vivo*. The relative importance of the level of endogenous extracellular dopamine in the brain and the degree of *in vitro* D_3_Rs receptor affinity and selectivity as determinant factors for *in vivo* D_3_Rs occupancy by antipsychotics, are also discussed.

## Introduction

It is widely accepted that dysfunction of the dopaminergic neurotransmitter system plays a major role in the pathophysiology of schizophrenia. The primary pharmacotherapy of schizophrenia involves the use of antipsychotics, a group of drugs representing great heterogeneity in their chemical structure, pharmacological and functional profile, as well as clinical efficacy. At present, all available antipsychotics display affinity for D_2_Rs, and it is widely accepted that D_2_R antagonism or partial agonism is essential for their antipsychotic efficacy. Currently used antipsychotics display medium-to-high *in vitro* affinity for D_2_R as well as D_3_R, and high correlation can be demonstrated between their affinities for these receptors. This is not surprising considering the high structural homology, and the *in vitro* functional and pharmacological similarities of the two receptors. On the other hand, significant differences have been demonstrated in their *in vivo* pharmacology and behavioral roles. All currently used antipsychotics, in agreement with their *in vitro* D_2_R affinity, show significant *in vivo* brain D_2_R occupancy at their antipsychotic effective doses. However, despite their substantial *in vitro* D_3_R affinity, not all antipsychotics demonstrated *in vivo* D_3_R occupancy in animals or in humans. Here, a review is given on the data available for the *in vitro* affinity for D_2_Rs and D_3_Rs and a hypothesis is provided as to why a group of antipsychotics do not show significant *in vivo* brain D_3_R occupancy despite their notable *in vitro* D_3_R affinity.

## Schizophrenia

Schizophrenia is one of the most serious and debilitating psychiatric disorder affecting about 1% of the population disregarding economic, social, or cultural background of the society (1). Schizophrenia is characterized by positive symptoms (delusions, hallucinations) negative symptoms (social and emotional withdrawal, anhedonia, lack of motivation) and cognitive dysfunction, as well. All these symptoms may be mixed with aggressive behavior, depression, or anxiety (2–4).

The early, so called “dopamine hypothesis” stated that low prefrontal dopamine activity would cause “deficit symptoms” whereas enhanced activity in mesolimbic dopamine system would be in the background of the positive symptoms (5). In fact, the increased dopamine transmission has been demonstrated by positron emission tomography (PET) (6, 7). Further, presynaptically increased synthesis of dopamine in the basal ganglia has been found [(8, 9), see for review]. Loss of glutamatergic functions is also hypothesized and is thought to explain negative symptoms (9–11).

## Antipsychotics

Recognition of the neuroleptic action of chlorpromazine in 1952 represented a breakthrough in the drug treatment of schizophrenia (12). Chlorpromazine was soon followed by introduction of several other “neuroleptics” such as haloperidol, fluphenazine, pimozide, sulpiride, thioridazine etc. (Interestingly enough, this group of drugs was named/categorized by their side effect profile).

At the time of their discovery, the main mechanism of action of the first-generation antipsychotics was believed to be mediated by their actions on the monoaminergic system. Carlsson and Lindquist demonstrated that haloperidol and chlorpromazine increased monoamine turnover in the rat brain and these changes were attributed to the monoamine receptor antagonism action of these compounds (13). Van Rossum was the first describing that antipsychotics exert their therapeutic effects through the blockade of dopamine receptors (14). For the history of antipsychotics’ discovery see the recent review by Seeman (15).

Some antipsychotics, such as clozapine, fluperlapine and melperone were found to produce weak catalepsy in rodents, with minimal extrapyramidal symptoms and serum prolactin elevation in humans, compared to the earlier typical antipsychotic drugs, such as haloperidol. Meltzer and Matsubara explored the basis of these differences by testing the affinity of 38 antipsychotics for the rat striatal dopamine D_1_ receptors (D_1_Rs), D_2_R and serotonin 5-HT_2_ receptors (5-HT_2_R). They found that the 5-HT_2_R/D_2_R affinity ratio was the most useful means of differentiation from the typical antipsychotics. They demonstrated that compounds displaying 5-HT_2_R/D_2_R affinity ratio of 1.12 or higher were the ones showing the atypical characteristics (16). These findings had significant impact on the antipsychotic drug research: the primary aim was to find antipsychotics possessing a significant serotonin 5-HT_2A_ receptor (5-HT_2A_R) affinity that would be similar or higher than that for the D_2_R. The quest for compounds with D_2_R/5-HT_2A_R affinity led to discovery of risperidone, asenapine, olanzapine, quetiapine, ziprasidone, blonanserin and lurasidone, collectively classified as atypical or second-generation antipsychotics.

Atypical antipsychotics, like to the typical antipsychotics, are efficacious in the treatment of positive symptoms of schizophrenia but display relatively lower propensity to cause extrapyramidal side effects. However, it was claimed that the label of “atypical” is not fully justified as they are different from first-generation antipsychotics only in their side effect profile (e.g., weight gain, alteration in metabolic parameters, cardiovascular complications) (17–19). In fact, neither group represented major step forward in the treatment of other symptoms of schizophrenia, such as negative or cognitive symptoms.

Distinct category of second-generation antipsychotics with partial agonism at dopamine D_2_R, D_3_R and serotonin 5-HT_1A_ receptors (5-HT_1A_R) as well as antagonism at serotonin 5-HT_2A_R and 5-HT_2B_ receptors (5-HT_2B_Rs) is represented by aripiprazole, cariprazine and brexpiprazole. Amongst these three partial agonist antipsychotics, aripiprazole and brexpiprazole display preferential binding affinity for dopamine D_2_R (20, 21), whereas cariprazine has higher affinity for dopamine D_3_R over D_2_R receptors (22). These dopamine receptor partial agonists may be referred to as third generation antipsychotics (23). These dopamine-serotonin partial agonists were originally approved for acute schizophrenia, schizophrenia maintenance, later, however, they were found to be useful in treatment of mania, bipolar disorder, and as adjunct in unipolar depression (24).

## Dopamine Receptors

Effects of dopamine are mediated through five receptors subtypes, namely D_1_-, D_2_-, D_3_-, D_4_-, and D_5_-receptors. All dopamine receptors belong to G-protein coupled receptor (GPCR) family: D_1_ and D_5_ receptors (D_1_-receptor family) stimulate cAMP signaling pathway through a G_α*s*_ G-proteins, whereas D_2_-, D_3_- and D_4_-receptors (D_2_-receptor family) inhibit cAMP signaling through a G_α*i/o*_ G-proteins (25–29).

Expression of dopamine D_1_ receptors (D_1_R) is the highest in basal ganglia (caudate nucleus, putamen and globus pallidus), accumbens nuclei, substantia nigra, amygdala and the frontal cortex. The cortex, substantia nigra, hypothalamus and the hippocampus express low level of dopamine D_5_ receptors (D_5_Rs). High levels of D_2_Rs are found in the basal ganglia, while cortical regions express low level of these receptors. D_2_Rs are the primary drug targets in schizophrenia, Parkinson’s disease, restless leg syndrome and neuroendocrine tumors. Highest expression of dopamine D_3_Rs are found mainly in the limbic system (islands of Calleja, nucleus accumbens, ventral part of caudate nucleus), with minor/low levels of expression in cortical regions. Dopamine D_4_ receptors (D_4_Rs) are found with relatively low level of expression in the amygdala, hippocampus, hypothalamus, cortex and, in the substantia nigra (25–28, 30–34).

## D_2_Rs as Key Targets for the Therapeutic Action of Aps

### *In vitro* Affinity and Selectivity of Antipsychotics for Dopamine Receptors of D_2_R-Subtype

First and second-generation antipsychotics possess diverse structural, pharmacological (*in vitro* receptor profile, functional activity, e.g., antagonism, partial agonism, inverse agonism) and behavioral effects and side-effect profiles. However, their common property is that all display medium-to-high affinity for dopamine receptors of D_2_R-subtype (i.e., D_2_R, D_3_R, and/or D_4_R) under *in vitro* conditions (18, 35–40). The *in vitro* affinities of currently used antipsychotics for dopamine D_2_R-like (i.e., D_2_R, D_3_R, and D_4_R subtypes) and their degree of D_3_R selectivity are summarized in Table 1.

**TABLE 1:** *In vitro* affinity of major first-, second-, and third-generation antipsychotics at human dopamine receptors of D_2_R-type and their degree of their D_3_R selectivity.

Compound	Ki (nM)			D_3_ selectivity	
	D_2_R	D_3_R	D_4_R	vs. D_2_R	vs. D_4_R
Amisulpride	3.0	2.4	2,369	1.3	984
Aripiprazole	0.9	1.6	514	0.56	321
Asenapine	1.4	1.8	1.8	0.78	1
Blonanserin^I^	0.28	0.28	n/a	1	–
Brexpiprazole^II^	0.3	1.1	6.3	0.27	5.7
Cariprazine^III^	0.49	0.09	>1,000	5.8	>1,000
Chlorpromazine	2	3	24	0.67	8
Clozapine	431	283	39	1.5	0.14
F17464^IV^	12.5	0.12	>1,000	104	>1,000
Fluphenazine	0.5	0.7	36	0.71	51
Haloperidol	2.0	5.8	15	0.34	2.6
Iloperidone	0.4	11	13.5	0.04	1.2
Loxapine	10.0	23.3	12	0.43	0.52
Lurasidone^V^	1.0	15.7	29.7	0.06	1.9
Lumateperone^VI^	32	n/a	n/a	n/a	–
Olanzapine^VII^	21	34.7	19	0.6	0.50
Paliperidone	9.4	3.2	54.3	2.9	17
Quetiapine^VII^	417	383	1,202	1.1	3
Risperidone^VII^	6.2	9.9	18.6	0.6	0.33
Ziprasidone	4.0	7.4	105	0.54	14
Zotepine	25	6.4	18	3.9	2.8

*I: (41); II: (21); III: (22); IV: (42); V: (43); VI: (44); VII: (39). n/a, no data available. A part of affinity data were taken from Ellenbroek an Cesura (37), and the PDSP data base (https://pdsp.unc.edu/pdspweb). The same data base-derived data for major antipsychotics are given in Gross and Drescher (38) and Kaar et al. (40), however, the affinities were somewhat different even though they were taken from the same data base. Receptor affinity data for major antipsychotics generated by Tadori et al. (20), Seeman (35), and Shahid et al. (39) also differed from the above data-based sources.*

### Daily Dose and Plasma Levels of Antipsychotics Correlates With Their *in vitro* Affinity for Dopamine D_2_Rs

Seeman demonstrated a close correlation between the therapeutic doses of antipsychotics and their *in vitro* D_2_R receptor affinity, but no correlation was found with D_1_R affinity (45, 46). Correlation between D_2_R affinities, optimal occupancy of brain D_2_R for antipsychotic efficacy (i.e., 60–70%) and the free plasma levels of antipsychotics were also demonstrated (47).

### Antipsychotics Occupy D_2_Rs in Brain

At present, it is broadly accepted that D_2_R affinity is the primary mechanism for antipsychotic efficacy (18, 36, 48, 49). Positron emission tomography (PET) studies demonstrated that for the clinical efficacy of D_2_R antagonist antipsychotics, a 60–75% occupancy of brain D_2_R is essential (50). In case of partial agonist antipsychotics, such as aripiprazole or cariprazine D_2_R occupancy can be as high as 95% at dose levels with established clinical efficacy (51–53), whereas brexpiprazole produced only 80% occupancy at the highest dose applied (54).

At present, despite the great efforts to develop non-dopamine antipsychotics, no such compounds are approved for the treatment of positive, negative, or cognitive symptoms of schizophrenia (55).

## D_3_R, a Potential Novel Target in the Therapy of Central Nervous System Disorders: Comparison With D_2_R

### Similarities and Differences of D_2_Rs and D_3_Rs

#### Structural

The D_3_R is a member of the largest phylogenetic class of GPCRs, known as class A, which contains the transmembrane domain without a large extracellular domain. Native ligands of aminergic GPCRs bind directly to the transmembrane domain, which is composed of seven transmembrane (TM) helices embedded in the cell membrane connected by three extracellular (EL) and three intracellular (IL) loops (56). The C-terminus of the protein is the eighth small α-helix (H8).

**FIGURE 1:**
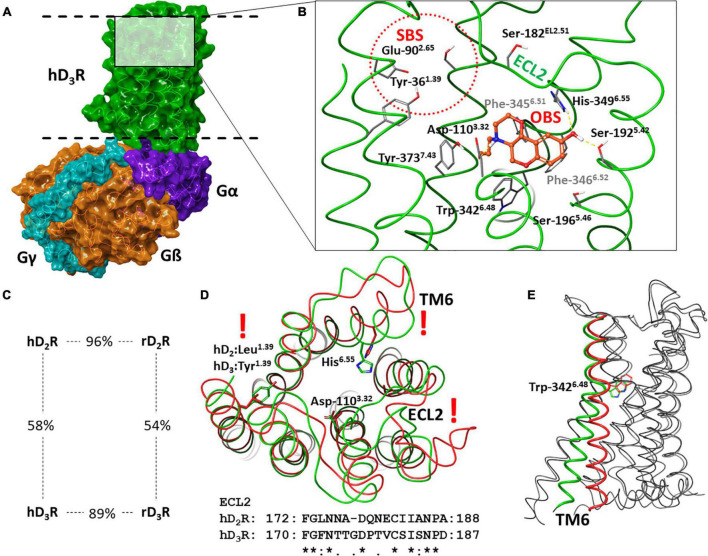
Structure of hD_3_R: **(A)** hD_3_R-G_*i*_ complex; **(B)** binding of the selective agonist PD128907 in the orthosteric binding site (Secondary binding site and ECL2 are also depicted); **(C)** percent identity of human and rat D_2_R and D_3_R sequences; **(D)** comparison of hD_2_R (red) and hD_3_R (green); **(E)** comparison of active (green) and inactive (red) conformation of hD_3_R*.

Analysis of amino acid sequence of human and rat dopamine D_2_R and D_3_R exhibits a high level of general sequence identity which is increased in the transmembrane helices forming a highly conserved orthosteric binding site (OBS) (see Figures 1A–C). The most obvious differences in the sequences can be found in the intracellular loop region (ICL3) between transmembrane helices of the TM5 and TM6. However, this region is quite distant from the orthosteric binding site, and thus the differences in the ECL2 (between the TM4 and TM5) and in the secondary binding site (SBS) are more relevant for the discovery of selective D_3_R vs. D_2_R ligands (57, 58). Moreover, targeting SBS may be a tool for fine tuning functional activity and biased agonism (59, 60). The shape and the sequence of the ECL2 is highly different in D_2_R and D_3_R (see Figure 1D). The SBS is the most probable binding site for the tail group of several elongated D_3_R ligands, where for instance the amino acid at the position 1.39 [Ballesteros-Weinstein numbering; (61)] is leucine in the D_2_R and tyrosine in the D_3_R. The amino acids forming the OBS are identical, but comparison of D_2_R and D_3_R structures suggest a slightly different shape of OBS because of the slightly different TM6 orientation (62).

Recently published experimental structures of D_2_R and D_3_R (62–68) provide extensive information sources on ligand binding and receptor function. Like other GPCRs, the most conspicuous change during activation is the movement of the TM6, which enables the G-protein to connect to the receptor (see Figure 1E). The Trp in the position 6.48 may have a key role in the activation since it is close to the OBS and its position is related to the TM6 orientation (62).

#### Intracellular Signaling Pathways

All dopamine receptors belong to GPCR family: D_1_R and D_5_R receptors (D_1_-receptors family) stimulate cAMP signaling pathway through G_α*s*_ G-proteins whereas D_2_R, D_3_R, and D_4_R (D_2_R family) inhibit this pathway through G_α*i/o*_ G-proteins. There exists cAMP- independent pathways such as the recently recognized ß-arrestin pathway which is thought to be involved in several physiological functions and drugs’ effects (25–29).

Upon activation, both isoforms of D_2_R (i.e., D_2_Short and D_2_Long) and D_3_R inhibit the enzyme adenylyl cyclase (AC) through G_α*i/o*_ subtype of G-protein leading to inhibition of cAMP-PKA-pDARPP32-PPI pathway. However, differences may exist in the coupling efficiency of the two receptors and AC (or its subtypes).

In different cell lines, both D_2_R and D_3_R can activate ERK/MAPK signaling albeit with different mechanisms: D_2_Rs are coupled to and activate through α-subunit of G_*i/o*_ protein following agonist stimulation whereas, D_3_R functions through G_*o*_ or G_ß_ subunit depending on the signaling machinery of the given cell line. Both D_2_Rs and D_3_Rs are positively coupled to ß-arrestin-Akt-GSK3 pathway. GSK3ß is expressed in several brain regions and plays important role in neuronal development, neurovegetative and psychiatric diseases such as schizophrenia or bipolar disorder (26, 29, 70–79).

#### *In vitro* Pharmacological Profile of Dopaminergic Agents at D_3_Rs vs. D_2_Rs

**FIGURE 2:**
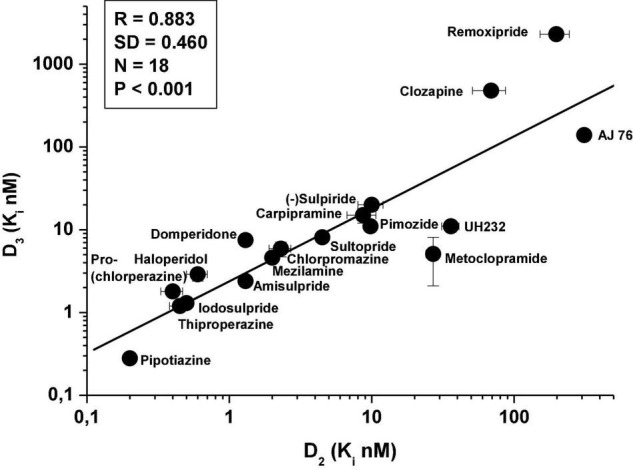
Correlation between *in vitro* affinity of various dopaminergic antagonists for human recombinant D_3_Rs and D_2_Rs [data taken from Sokoloff et al. (80)]; ligand: [^125^I]sulpiride.

It has been demonstrated that significant correlation exists between the *in vitro* affinities of various dopaminergic agents (agonists, antagonists, partial agonists) for D_2_Rs and D_3_Rs (80) (Figure 2).

**FIGURE 3:**
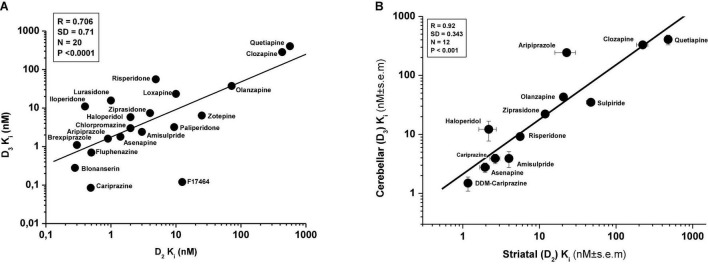
Correlation between *in vitro* affinity of antipsychotics for human D_2_Rs and D_3_Rs (**A**, data taken from Table 1; various radioligands) and for rat striatal D_2_Rs (striatal membrane) and cerebellar D_3_Rs (CB L9,10 membrane) **(B)**; rat D_2_R and D_3_R affinity data derive from the extension of the study of Kiss et al. (81) and Kiss et al. (82). Determination of D_2_R and D_3_R affinity in membranes from CHO cells expressing human D_3_R or cerebellar L9,10 membranes (ligand: [^3^H]-(+)-PHNO) is described in Kiss et al. (81).

Further results, using additional compounds, have confirmed earlier evidence showing close correlation between affinities of antipsychotics for human recombinant D_2_Rs and D_3_Rs (Figure 3A). However, no such correlation was found between D_1_R vs. D_3_R or D_3_R vs. D_4_R (data not shown). Similarly, high level of correlation was found between the affinity of antipsychotics for the rat D_2_R and D_3_Rs using [^3^H](+)-PHNO radioligand (81, 82) (Figure 3B).

Based on recognition that D_3_Rs are mainly expressed in the limbic system (*vide supra*), the region is involved in schizophrenia pathology, and that significant correlation existed between the affinity of antipsychotics for D_2_Rs and D_3_Rs, it was thought that D_3_R affinity may play a role in the therapeutic efficacy of antipsychotics and led to propose development of selective D_3_R antagonists as novel antipsychotics (30, 80, 83–85).

#### Predicted Binding Mode of Antipsychotics in the D_3_R

One of the available experimental structure studies of D_3_R has been carried out with the antagonist eticlopride (63), and the other two with the agonists, pramipexole and PD128907 (62). All these agents bind to the orthosteric binding site (see Figure 1B). The most important interactions are the salt bridge with the Asp-110^3.32^ as well as the π-π interactions with the aromatic residues (e.g., Trp-342^6.48^, Phe-345^6.51^, Phe-346^6.52^), which form a lipophilic cavity. Hydrogen bond interaction with the serines in the 5.42 and 5.46 positions is typical for agonist binding state in D_3_R (62), and also in D_2_R structures (64, 65).

Non-selective ligands most probably bind to both the D_2_R and D_3_R in the same binding mode, forming a very similar interaction pattern. Thus, the D_2_R structural binding results obtained for non-selective D_2_R/D_3_R antagonists, such as risperidone, haloperidol or spiperone can be predictive of their binding mode at the D_3_R. It should be noted that distinct inactive conformations of D_3_R exists, and ligands may have different preferences which lead to different functional behaviors of antagonists (antagonism vs. inverse agonism, sensitivity for sodium ions) (86). These results are in line with the well-known highly dynamic nature of the GPCRs (87).

**FIGURE 4:**
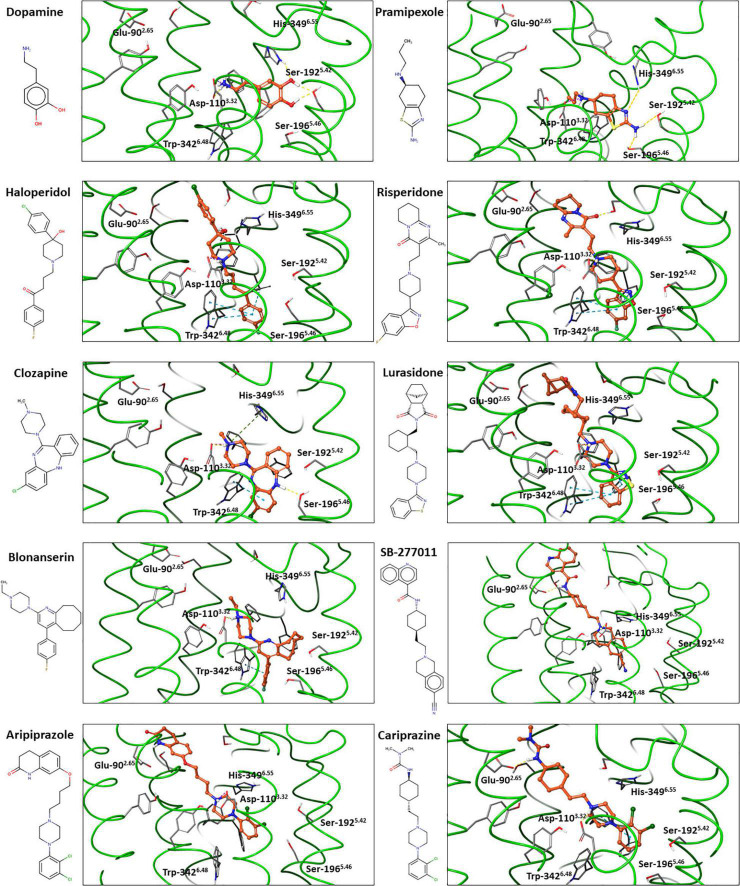
Dopamine, pramipexole and selected antipsychotics docked into experimental D_3_R structures*.

Based on the available experimental structural information supplemented by computational investigations (60, 88–90) the binding mode of antipsychotics at the D_3_R can be predicted at a reliable manner. In order to illustrate this, we docked several selected ligands into the D_3_R structures available in the Protein Data Bank (PDB ID: 7CMV (62) for dopamine and 3PBL (63) for the others) using the Glide, induced-fit-docking and the protein-ligand complex refinement protocols implemented in the Schrödinger software package (Schrödinger Release 2020-2) (Figure 4).

#### *In vivo* Roles and Behavioral Pharmacology of D_3_R and D_2_Rs Is Different

Despite the similarities in the *in vitro* properties of D_3_Rs and D_2_Rs described above, the *in vivo* roles and behavioral pharmacology of D_3_Rs compared to D_2_Rs are remarkably different. Animal data suggest opposite role of D_2_R vs. D_3_R in the control of locomotor activity, and cortical functions such as learning and memory (91, 92). On the other hand, both D_2_R and D_3_R receptor agonists were shown to impair certain social functions and cognitions (93–95). Enhanced expression of striatal dopamine D_3_R receptors impairs motivation (96). Antagonists of dopamine D_2_R receptors stimulate prolactin secretion (18), whereas D_3_R antagonism does not produce such effect either in rats or in human (97, 98). Majority of D_2_R antagonist antipsychotics (e.g., haloperidol, risperidone, and olanzapine) elicit catalepsy at higher doses (99). In contrast, D_3_R antagonists do not cause catalepsy (97), they rather inhibit haloperidol-induced catalepsy (100, 101).

Microdialysis studies demonstrated that D_2_R antagonist antipsychotics enhance, whereas selective D_3_R antagonists (such as SB-277011) (97, 102) or D_3_R-preferring D_3_R/D_2_R (such as S33138) antagonists (84, 103) exert no or minimal effects on cortical or striatal dopamine release (104).

Little is known on the functions of dopamine D_3_R receptors in humans although their involvement is assumed in central nervous system (CNS) diseases such as schizophrenia, Parkinson’s disease, addiction, anxiety, and depression or in the clinical effects of antipsychotics (26, 38, 70, 75).

## Selective Agonists or Antagonists for D_3_R: the Challenge of Drug Research

The availability of drugs displaying high selectivity and affinity for D_2_R or D_3_R receptors are of great importance. Such compounds are useful tools in the exploration of neural mechanisms related to dopamine D_3_R receptors and may lead to novel agents for the treatment of various CNS disorders. Because of the close similarity in structure and signaling pathways of D_2_R and D_3_R, development of highly selective compounds for either subtype has been very challenging (34, 105).

Amongst agonists, the *in vitro* D_3_R affinity and selectivity of 7-OH-DPAT, PD128907 and pramipexole demonstrated great variability depending on the assay conditions used (105). Nevertheless, their degree of D_3_R vs. D_2_R selectivity seems adequate for use as tools for *in vitro* studies and their *in vivo* D_3_R selectivity may not be optimal, as they may also stimulate D_2_Rs within a narrow dose range (38, 106–109). For example, all three compounds produce biphasic behavioral effects in rats, some of which can be inhibited by either D_3_R and/or D_2_R selective antagonists, depending on the exposure levels of these agonists (95, 110–114).

The quest for high affinity, selective antagonists for D_3_R receptors (i.e., low-nanomolar K_*i*_ with D_2_R/D_3_R selectivity ≥100) began soon after the discovery of D_3_R. Several antagonists fulfilling the selectivity requirements such as SB-277011A (97), ABT-925 (115), GSK598809 (116), compound 74 in Micheli et al. (117) are currently available for experimental purposes. The pharmacological properties of the selective D_3_R antagonists have been reviewed by Gross et al. (84). L-741626 seems to be relatively selective for D_2_R reaching 100-fold higher D_2_R affinity vs. D_3_R, depending on the assay system used (118).

## Selective D_3_R Antagonists as Antipsychotics?

Compounds with relatively high selectivity for dopamine D_3_Rs such as SB-277011A (97), S33084 (119), ABT-925 (115, 120), GSK598809 (116, 117), or the D_3_R-preferring D_3_R/D_2_R antagonist S33138 (103), or the D_3_R-preferring partial agonist BP-897 (121) demonstrated antipsychotic-like properties in animal models, however none of them reached therapeutic application. The high affinity D_3_R -preferring antagonist F17464 with partial agonism at serotonin 5-HT_1A_R and antagonism at dopamine D_2_R (42) showed promising preclinical profile as well as clinical efficacy in schizophrenia patients in a Phase II study. This compound is still under development and (122, 123). Propose the development of selective D_3_R antagonist for the treatment of negative symptoms of schizophrenia based on the available scientific evidence (84).

## Imaging the D_3_Rs *In Vivo*

### PHNO for Labeling D_3_Rs

Number of tracers have been tried to develop for selective imaging of D_3_Rs in the brain (124–126), however, the only radioligand currently available for labeling of D_3_R in occupancy studies suitable for separation of D_3_R and D_2_R signal is the [^3^H]- or [^11^C]-labeled (+)-4-propyl-9-hydroxynaphthoxazine [(+)-PHNO, naxagolide]. (+)-PHNO was originally described as an orally acting, potent dopamine receptor full agonist (127). (+)-PHNO was shown to possess 50-fold selectivity for human recombinant D_3_R (K_*i*_: 0.16 nM) vs. D_2_R (Ki: 8.5 nM) (128).

[^11^C]-(+)-PHNO was synthesized by Wilson et al. (129) and it was shown that, in contrast with the antagonists such as [^11^C]raclopride, [^18^F]Fallypride, [^11^C]FLB-457 or the agonist [^11^C]N-methyl-norapomorphine, (+)-PHNO highly binds to regions rich in D_3_Rs. Using selective compounds such as the D_3_R antagonists SB-277011A, GSK598809 or the D_2_R antagonist SV-156, [^11^C]-PHNO proved to be useful for the separation of D_3_R and D_2_R binding signal and quantification of D_3_Rs in the brain, thus becoming an important tool for the investigation of the *in vivo* D_3_R occupancy by antipsychotics (116, 129–136).

### D_3_R Occupancy of Antipsychotics—Animal Studies With [^3^H](+)PHNO

It was reported that after *intravenous* administration of [^3^H](+)-PHNO, D_3_Rs are labeled in the rat cerebellum L9,10 and D_2_R in the striatum. This is based on the finding that the selective D_3_R antagonist, SB-277011 inhibited [^3^H](+)-PHNO binding in CB L9,10 membranes but not in the striatum whereas, the opposite profile was obtained with the D_2_R selective antagonist, SV-156 (118) (compound 9); (81).

Using the above approach, olanzapine, risperidone, haloperidol, and clozapine given acutely or chronically, at doses corresponding to human doses, showed nearly full occupancy in the striatum and NAC (D_2_R rich regions) with significantly lower level or no occupancy in VP, ICj and substantia nigra (SN) (D_3_R rich regions). In contrast, in the *in vitro* autoradiography experiments all these antipsychotics inhibited [^3^H]-(+)-PHNO binding in the above regions except CB L9,10. It was concluded that under *in vivo* conditions the above-mentioned antipsychotics occupy dopamine D_2_R but not D_3_Rs despite their significant affinity for D_3_Rs *in vitro* (137, 138).

We extended this approach and compared the *in vitro* affinity of several dopamine D_2_R/D_3_R agonists, partial agonists, and antipsychotics using membranes prepared from rat striatum (D_2_R-rich) and cerebellar L9,10 region (D_3_R rich) to determine their *in vivo* D_3_R and D_2_R occupancy. The affinity data are given in Kiss et al. (82). We also compared the effects of systemic administration of selected full agonists, partial agonists and antipsychotics on the *in vivo* binding/uptake of intravenously given [^3^H](+)-PHNO binding/uptake in the rat striatum and cerebellar L,910 regions. The results are summarized in Table 2. Among the drugs with subnanomolar or low nanomolar Ki values for D_3_R, full agonists pramipexole and PHNO potently inhibited [^3^H](+)-PHNO binding of CB L,910 membranes with marked preference toward CB L9,10 D_3_Rs. Cariprazine, didesmethyl-cariprazine (DDCAR), asenapine, raclopride and amisulpride, produced dose-dependent inhibition of [^3^H](+)-PHNO binding/uptake both in the striatal and CB L9,10 regions. Raclopride and asenapine, however demonstrated high striatal vs. cerebellar selectivity (82). The antipsychotics, aripiprazole, olanzapine, risperidone, quetiapine, ziprasidone (all with high nanomolar K_*i*_ values) produced inhibition of [^3^H](+)-PHNO binding/uptake in the striatum and little or modest level of inhibition in the CB L9,10.

**TABLE 2:** Effects of selected antipsychotics, D_3_R agonists, antagonists, on the [^3^H](+)-PHNO uptake in rat striatum and cerebellum L9,10 region^*,&^.

	Route	Administered highest dose (mg/kg)	Striatal ED_50_ (mg/kg)	CB L9/10 ED_50_ (mg/kg)	Striatum/ CB L9,10 ratio
**Agonists**					
(+)-PHNO	p.o.	1	>1 (33)	0.05 (95)	>>20
(−)-Pramipexole (PRP)	s.c.	1	>1 (39)	0.018 (96)	>>55
**Partial agonists**					
Aripiprazole (ARP)	p.o.	30	7.65 (92)	>30 (14)	<<0.26
Cariprazine (CAR)	p.o.	3	0.23 (99)	0.43 (99)	0.53
Cariprazine	i.v.		0.023 (94)	0.035 (98)	0.66
DD-CAR^+^	p.o.	10	0.58 (99)	0.41 (100)	0.66
**Antagonists**					
Amisulpride (AMS)	i.p.	30	>30 (35)	3.0 (82)	>10
Asenapine (ASN)	s.c.	1	0.037 (95)	0.177 (74)	0.21
Clozapine^#^ (CLZ)	p.o.	60	60 (34)	60 (29)	n.c.
Haloperidol (HP)	p.o.	3	0.23 (100)	1.05 (100)	0.22
Olanzapine (OLZ)	p.o.	30	1.46 (91)	∼30 (48)	∼0.05
Quetiapine^#^ QUET)	p.o.	250	250 (36)	250 (36)	n.c.
Raclopride (RCP)	s.c.	1	0.013 (98)	0.072 (97)	0.18
Risperidone (RSP)	p.o.	3	0.29 (89)	∼2.3 (53)	∼0.13
SB-277011A (SB)	p.o.	30	>30 (28)	1.31 (100)	>>23
SV-156	s.c.	10	0.89 (84)	>12 (20)	<<0.07
Ziprasidone (ZPR)	p.o.	30	1.63 (92)	∼30 (52)	∼0.05

*The ED_50_ doses were calculated from individual dose response curves consisting of at least 4–5 doses with 3–8 animals in each dose-group. Group means were analyzed by one-way analysis of variance (ANOVA) followed by Tukey-Kramer post-hoc multiple comparison test. The highest inhibition percentage achieved at the highest applied are given in the brackets. Where the highest achieved inhibition at highest applied dose was around 50% percent, approximate ED_50_ values are given and are marked with ∼ sign.

*^+^DD-CAR, didesmethyl-cariprazine; one of the major human metabolites of cariprazine.*

*^#^In case of clozapine and quetiapine the highest achievable inhibition was less than 50%, thus ED_50_ could not be calculated.*

*^&^Kiss et al. (82).*

Blonanserin, an antipsychotic marketed in Japan, was originally described as D_2_R and serotonin 5-HT_2_R antagonist (139). It has recently been found that blonanserin displayed high affinity *in vitro* for human D_2_R and D_3_Rs (Ki: 0.28 nM). Using the *in vivo* [^3^H](+)-PHNO method it caused dose-dependent, high occupancy of striatal D_2_R and D_3_R in the rat CB L9,10. In agreement with our data (see above) risperidone, olanzapine and aripiprazole demonstrated high occupancy only in the striatum and moderate or no occupancy was noted in the CB L9,10 region (41).

### D_3_R Occupancy of Antipsychotics—Human PET Studies

In patients suffering from schizophrenia, occupancy of D_2_Rs and D_3_Rs following long-term treatment with risperidone, clozapine or olanzapine was examined using [^11^C]raclopride or [^11^C](+)-PHNO PET. This study demonstrated that these antipsychotics caused high D_2_R occupancy in the D_2_R-rich dorsal striatum, using either [^11^C]raclopride or [^11^C](+)-PHNO. However, they failed to show binding signal in the D_3_R-rich globus pallidus using [^11^C](+)-PHNO (140). Similar results with [^11^C](+)-PHNO PET were reported by Mizrahi et al. demonstrating that in drug-naive, first episode schizophrenia patients, olanzapine and risperidone resulted in high occupancy in the D_2_R-rich regions but not in the globus pallidus where even “negative occupancy” was noted (141). On the other hand, blonanserin, (hD_2_R Ki: 0.284 nM; hD_3_R Ki: 0.277 nM), in agreement with data obtained in rats, achieved significant D_3_R occupancy in healthy volunteers (142).

PET studies in healthy volunteers using [^11^C]raclopride (51) as well as in patients with schizophrenia using [^18^F]Fallypride (52) aripiprazole with D_2_R preference showed dose-dependent occupancy in the D_2_R-rich striatum without causing extrapyramidal side effects. A subsequent study with D_3_R preferring PET ligand, [^11^C](+)-PHNO confirmed the D_2_R occupancy of aripiprazole however, minor levels of D_3_R occupancy was detected (143).

Cariprazine, a D_3_R preferring D_3_R/D_2_R partial agonist antipsychotic (hD_2_R Ki: 0.49 nM; hD_3_R Ki: 0.09 nM) (22) dose-dependently inhibited [^11^C](+)-PHNO binding in brain regions with varying D_2_R and D_3_R expression. It showed significant occupancy of both D_2_R and D_3_R, albeit with approximately 3–6-fold selectivity for D_3_R (53, 143).

Brexpiprazole is also a partial agonist antipsychotic with D_2_R preference (hD_2_R Ki: 0.3 nM; D_3_R Ki: 1.1 nM) (21). Occupancy study in healthy volunteers showed that in the therapeutic dose range (1 and 4 mg/d) it produced only very low levels (i.e., 2–13%) of D_3_R occupancy whereas it achieved 36 and 59% D_2_R occupancy, respectively, in the applied dose range (54).

F17464 with remarkable affinity for D_3_Rs (D_3_R Ki: 0.16 nM; D_2_R Ki: 12 nM) demonstrated antipsychotic-like activity in animal experiments (42, 144). It was reported that in a double blind, multicenter Phase II study, F17464 (20 mg/bd) improved schizophrenia symptoms (122). In a phase I study, F17464 resulted in 69–95% occupancy of D_3_Rs whereas only a 20% occupancy of D_2_Rs were noted (145).

## Potential Explanation for Why Significant *In Vitro* Affinity May Not Guarantee Substantial D_3_ Occupancy *In Vivo*

### Role of Endogenous Dopamine

#### Affinity of Dopamine for D_3_R

The dopamine displays considerably higher *in vitro* affinities for D_3_Rs (K_*i*_ values vary from 30 nM to 100 nM) compared with D_2_R (K_*i*_ values vary from 200 nM to 1000 nM)^1^ ; (70). The *in vitro* K_*i*_ values greatly depend on several *in vitro* binding conditions such as the receptor source, radioligands used for binding assays, and assay methodology.

As to the dopamine K_*i*_ values for D_3_Rs the picture is further complicated since like D_2_R, D_3_R may also exist in low and high affinity state. Sokoloff et al. did not find differences between affinity of dopamine for D_3_R in the absence or presence of Gpp(NH)p (24 vs. 27 nM) (30, 80). However, Gross and Drescher (38) and Seeman et al. (146) reported remarkable difference between the low and high affinity states of D_3_R. D_3_Rs are prone to dimerization and to form heteromers with D_1_Rs or D_2_Rs, or with non-dopaminergic receptors (147). Affinity of dopamine (and the signalization pathway) as well as that of other dopaminergics (including antipsychotics) toward D_3_R di- or heteromers may also change.

#### Endogenous Dopamine Concentrations

As determined by *in vivo* microdialysis in rodents, under physiological conditions the extracellular, (i.e., resting or steady state) dopamine concentrations are in the low nanomolar range in various brain regions, including n. accumbens (∼1.5–4.5 nM), striatum (∼2–5 nM), hippocampus (∼1 nM) and in subnanomolar range in the prefrontal cortex (∼0.3–0.6 nM) (104, 148–154).

Little is known about the endogenous dopamine concentration in the human brain. Using [^11^C]-(+)-PHNO PET Caravaggio et al. estimated that the K_*d*_ (dissociation constant) of dopamine is 22–24 nM and they reported that concentration of dopamine is between 8 and 9 nM in the ventral striatum, caudate and putamen and 2.8 nM in the globus pallidus (155).

#### Endogenous Dopamine May Compete With Antipsychotics for Occupying D_3_Rs

Using *ex vivo* autoradiography Schotte et al. (156) demonstrated that endogenous dopamine had greater ability to occupy D_3_Rs as compared to D_2_Rs and concluded that D_3_Rs are preferably occupied by endogenous dopamine which “*limits the binding of antipsychotic drugs to D_3_ receptors in the rat brain*.”

The alkylating agent, EEDQ (1-ethoxycarbonyl-2-ethoxy-1,2-dihydroquinoline) concentration dependently reduced the *in vitro* [^3^H]7-OH-DPAT and [^3^H]spiperone binding in membranes from rat ventral striatum. *In vivo* treatment of rats with EEDQ resulted in reduction of the *ex vivo* [^3^H]spiperone binding in striatal membranes but did not alter [^3^H]7-OH-DPAT binding in membranes from ventral striatum. The author concluded [^3^H]7-OH-DPAT binding sites (i.e., mostly D_3_R) seem to be resistant to EEDQ-induced inactivation *in vivo* sites (157).

In agreement with these results, Zang et al. using autoradiography, demonstrated that treatment of rats with EEDQ or NIPS (*N*- *p*-iso-thiocyanatophenethyl-spiperone) did not alkylate D_3_Rs receptors in n. accumbens and in the island of Calleja at doses that resulted in blockade of D_2_Rs receptors in caudate and n. accumbens. On the other hand, under *in vitro* conditions when slices from the above regions were incubated with EEDQ or NIPS, both inhibited dopamine D_2_Rs as well as D_3_Rs and inhibition at D_3_R sites were prevented by dopamine in nanomolar concentration range whereas only millimolar concentration of dopamine was able to protect D_2_Rs. The authors concluded that their results *“are consistent with the view that alkylation of D_3_ receptors in vivo is prevented by its high affinity for even minor concentrations of endogenous dopamine*” (158).

### Modulation of Extracellular Dopamine by D_2_R Antipsychotic Treatment

#### Microdialysis Studies

The partial agonists antipsychotics such as aripiprazole (159, 160), brexpiprazole (21) and cariprazine (153, 154) caused only moderate or no change of the extracellular dopamine concentration in the rat prefrontal cortex, hippocampus, n. accumbens and in the striatum. It is interesting to note that the high affinity D_3_R-preferring antagonist antipsychotic, F17464 (K_*i*_ for D_3_R: 0.16 nM; Ki for D_2_R: 12.1 nM) also did not significantly change extracellular dopamine concentration in the medial prefrontal cortex (42).

Both the typical antipsychotic haloperidol and the atypicals such as such as asenapine, blonanserin, clozapine, risperidone, olanzapine, lurasidone, and ziprasidone dose-dependently and remarkably (by 2- to 4-fold) elevated the extracellular dopamine concentrations in the rat prefrontal cortex, hippocampus, n. accumbens and in the striatum (104, 148–152, 161, 162). It should be mentioned that the above antipsychotics, beside their D_2_R affinity, display high affinity for adrenergic alpha, dopamine D_3_, D_4_, serotonin 5-HT_2A_, 5-HT_1A_, 5-HT_2B_, 5-HT_6_, and 5-HT_7_, muscarinic, and histaminergic receptors (37) which may influence the extracellular dopamine levels evoked *via* D_2_R antagonism. In fact, among atypical antipsychotics risperidone, asenapine, increased extracellular concentration of serotonin in the prefrontal cortex (151, 161), while olanzapine (162), lurasidone (152) blonanserin (137), and clozapine (138) resulted in modest or no effect. Olanzapine, blonanserin, asenapine and haloperidol significantly increased extracellular norepinephrine levels, as well (137, 148, 163).

#### D_2_R Antagonists Directly Inhibit Dopamine Transporter

Former studies showed that D_2_R antagonists can inhibit dopamine uptake *via* D_2_Rs (164). Amato et al. have recently proposed that beside D_2_R antagonism/occupancy, the direct blockade of DAT by antipsychotics, i.e., the modulation of extracellular dopamine, is a likely important factor in the antipsychotic efficacy (165–167).

The involvement of D_3_Rs in the regulation of DAT or the effects of antipsychotics *via* D_3_Rs on the DAT is much less known. Zapata et al. found that D_3_R upregulate DAT (168), whereas Luis-Ravelo et al. demonstrated that the regulation appears to be biphasic, i.e., acute D_3_R activation increased DAT expression whereas prolonged activations reduced dopamine uptake (169).

#### Turnover Studies

Early studies found greatly increased dopamine turnover rate in the rat or cat brain after antipsychotic treatment (170–172).

We compared the effects of selected antipsychotics, D_3_R or D_2_R antagonists and D_3_R preferring dopamine agonists on the dopamine turnover index in the rat striatum (and in olfactory tubercle and n. accumbens, data not shown) with D_3_R occupancy ED_50_ doses (i.e., doses causing 50% inhibition of [^3^H](+)PHNO uptake/occupancy, Table 2) in the striatum and in CB L9,10.

**FIGURE 5:**
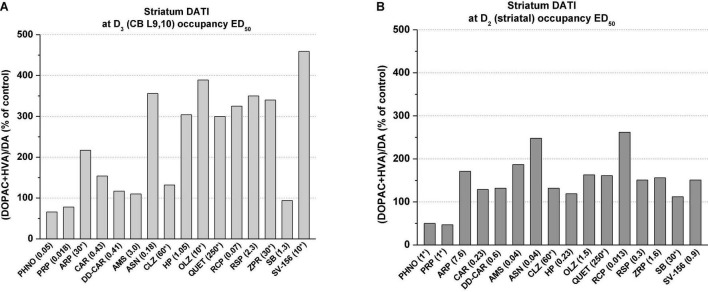
Graphical presentation of percent changes in striatal dopamine turnover indices (DATI) at D_3_R (i.e., cerebellar L9, 10) occupancy ED_50_ doses **(A)** and at D_2_R (i.e., striatal) occupancy ED_50_ doses **(B)** in vivo^*,#^. *Doses in brackets denote the occupancy ED_50_ doses (or close to ED_50_) taken from Table 2. Dopamine turnover index (DATI) was estimated from turnover dose-response curves (consisting of at least 4–5 doses, with five rats in each dose-group) for individual compounds listed in this figure. In cases where the occupancy ED_50_ values could not be exactly calculated (see Table 2) the turnover indices were determined at doses denoted with asterisks. Dopamine turnover index was defined as DA/(DOPAC+HVA). Determination of tissue dopamine, DOPAC and HVA was carried out exactly as described in Kiss et al. (22) (for abbreviations of drugs’ names, see Table 2). ^#^Dopamine turnover data for cariprazine and DD-CAR were published in Kiss et al. (173). Turnover results of other compounds are unpublished and are on file at G. Richter. Plc.

At cerebellar (i.e., CB L9,10 D_3_R) occupancy ED_50_ doses, the agonists (+)-PHNO and (-)-pramipexole reduced the striatal dopamine turnover index by about 50%, whereas antipsychotics such as asenapine, haloperidol, olanzapine, risperidone, and ziprasidone and the D_2_R preferring antagonist SV-156 greatly enhanced (by about 3–4-fold) striatal dopamine turnover index (Figure 5A). Blonanserin was not involved in this study, but it is reported that it caused 3–4-fold increase of striatal, frontal and limbic (i.e., olfactory tubercle and n. accumbens) DOPAC and HVA, which are all clearly indicate turnover increasing effect of blonanserin (139). The partial agonist cariprazine, the cariprazine metabolite, DD-CAR did not significantly change the striatal dopamine turnover index as was noted with amisulpride and the D_3_R antagonist SB-277011A. Interestingly enough, the D_2_R partial agonist aripiprazole produced effects more like to those seen with D_2_R antagonist antipsychotics.

On the other hand, at the D_2_R occupancy ED_50_ doses (i.e., doses causing 50% inhibition of striatal [^3^H](+)-PHNO uptake) which were much lower than that of necessary for 50% occupancy of CB L9, 10 D_3_Rs, all antipsychotics (i.e., asenapine, haloperidol, olanzapine, risperidone, and ziprasidone and the D_2_R preferring antagonist SV-156) caused much less increase in dopamine turnover index (Figure 5B). The effects of the partial agonist cariprazine, DD-CAR and the SB-277011A, at their D_2_R occupancy doses, produced modest turnover changes in the striatum as was seen at their D_3_R ED_50_ occupancy doses.

The results of dopamine turnover studies, in agreement with microdialysis results, indicate that D_2_R antagonist antipsychotics greatly increase the dynamics of dopamine metabolism including the increase of extracellular dopamine at doses sufficient to achieve occupancy of D_3_Rs. Opposite effects were seen with dopamine D_3_R-preferring agonists (-)-pramipexole and (+)-PHNO (which is probably due to the D_2_R agonist effects manifested under *in vivo* conditions). At pharmacological doses, neither cariprazine nor its one of the major metabolite, DD-CAR did not seem to alter significant alteration in dopamine metabolism in rat striatum.

### Affinity and/or Selectivity of Compounds for D_3_Rs *in vitro* vs. D_3_R Occupancy *in vivo*

In Table 3, a summary is given on the D_2_R and D_3_R affinity and selectivity of some D_3_R selective antagonist, agonists, and antipsychotics along with their D_3_R occupancy determined in rats or in human.

**TABLE 3:** Summary of *in vitro* human D_3_R affinity, D_3_R selectivity and occupancy* of some antipsychotics, partial agonists, highly preferring/selective D_3_R agonists, antagonists.

Compounds	hD_2_R K_*i*_ (nM)	hD_3_R K_*i*_ (nM)	hD_3_R selectivity	Species	D_3_R occupancy	References
**D_3_R preferring agonists**						
(+)-PHNO	0.35	0.17	2.2	Rat	YES	(82, 129)
(-)-Pramipexole	42	1.85	23	Rat	YES	(82, 140)
**Selective D_3_R antagonists**						
ABT-925	600	2.9	207	Human	YES	(98, 174)
GSK598809	740	6.2	119	Human	YES	(116)
SB-277011A	1047	11	95	Rat	YES	(82, 175, 176)
F17464	12	0.16	72	Human	YES	(145)
**Partial agonists**						
Aripiprazole	0.9	1.6	0.56	Rat, human	Low	(82, 143),
Brexpiprazole	0.3	1.1	0.27	Human	Low	(54)
BP897	61	0.92	66	Human	Moderate	(85)
Cariprazine	0.49	0.09	5.8	Rat, human	YES	(53, 82)
DD-CAR	1.41	0.056	25	Rat	YES	(173)
**Antipsychotics**						
Asenapine	1.4	1.8	0.78	Rat	YES	Table 2
Blonanserin	0.28	0.28	1	Rat, human	YES	(41, 142)
Clozapine	431	283	1.5	Rat, human	Low	(82, 140)
Haloperidol	2.0	5.8	0.34	Rat	YES	(82, 175)
Olanzapine	21	34.7	0.6	Rat, human	Low	(82, 141, 175)
Risperidone	6.2	6.9	0.9	Rat, human	Low	(82, 141, 175)
Quetiapine	417	389	1.1	Rat	Low	(82)
Ziprasidone	4.0	7.4	0.54	Rat	Low	(82)
**D_2_R antagonist**						
SV-156**	4.04	250	0.02	Rat	NO	(82)

*Rat or human brain occupancy determinations were carried out by [^3^H](+)-PHNO (rat) or [^11^C](+)-PHNO (human).

**Compound 9 in Vangveravong et al. (118).

Based solely on the *in vitro* affinity data one may expect compounds with low-or sub-nanomolar affinities for both receptor subtypes, would show D_2_R as well as D_3_R occupancy *in vivo*. However, the preclinical and human occupancy studies summarized above do not necessarily support such a correlation.

Both D_3_R-preferring agonist, (+)-PHNO and pramipexole as well as the antagonists (ABT-925, GSK598890, SB-277011A and the antipsychotic candidate F17464) all display low- or sub-nanomolar D_3_R affinity and high selectivity for D_3_Rs *in vitro*. These compounds produced D_3_R occupancy in rat or human studies. The same (i.e., high D_3_R affinity, selectivity *in vitro* and high D_3_R occupancy) is applicable for the partial agonists, cariprazine and its metabolite, DD-CAR. Although aripiprazole and brexpiprazole displayed low nanomolar *in vitro* D_3_R affinity, their D_3_R selectivity was below 1, which could explain their lack of D_3_R occupancy *in vivo*. Among the currently used antipsychotics, only the D_2_R/D_3_R antagonist blonanserin, which has low- or sub-nanomolar affinity for these receptors has been shown to have significant *in vivo* occupancy for both receptors in rats. Second generation antipsychotics (i.e., risperidone, quetiapine, clozapine) with low D_3_R affinity (K_*i*_: >3–10 nM) and selectivity resulted in negligible D_3_R occupancy.

## Limitations

Our knowledge about the occupancy of D_3_Rs in the rat or human brain comes from the use of [^3^H](+)-PHNO or the [^11^C](+)-PHNO radiotracers. Their use represented a great advance in the *in vivo* imaging of D_3_Rs and determination of occupancy of brain D_3_Rs by antipsychotics.

[^3^H](+)-PHNO or the [^11^C](+)-PHNO however, are not ideal ligands/tracers for several reasons. They may not be sensitive enough for more detailed mapping of D_3_Rs in regions having low D_3_R expression e.g., cerebral cortex. Although both display higher affinity than dopamine for D_3_R, they are still sensitive to endogenous dopamine (155, 177).

Furthermore, both D_2_Rs and D_3_Rs may exist in high- or low-affinity states and they are prone to di- or heteromerization (147, 178, 179). It was reported that recombinant human or rat D_3_R, like D_2_R, may exist in low- and high-affinity state and the affinity of PHNO shows significant difference for these states (30, 81, 128, 146, 180) which may have implication in drugs’ imaging studies (140, 155).

These conditions (i.e., the high/low affinity state and di- or heteromerization, if they exist) may greatly change the affinity of the two receptors toward the agonist tracer and the affinity of drugs to be examined and their occupancy. Thus, the quest for better ligands (agonist or antagonist?) for the demonstration of brain D_3_Rs occupancy *in vivo* by therapeutically useful compounds (e.g., antipsychotics among others) continues (109, 125, 126, 177, 181).

Moreover, in contrast with the known therapeutically optimal occupancy of antipsychotics at D_2_Rs (i.e., 65–75%) there is no reliable information on the optimal level of D_3_R occupancy for manifestation of therapeutic effect.

## Summary and Conclusion

All currently used antipsychotics display high-to-medium affinity for both D_2_R and D_3_Rs *in vitro*. In agreement with the *in vitro* D_2_R affinity they show significant D_2_R occupancy in the rat and human brain at their antipsychotic-effective doses. However, as revealed by animal and human occupancy studies, despite the considerable *in vitro* D_3_R affinity, not all antipsychotics demonstrated brain D_3_R occupancy *in vivo.*

There may exist several possibilities for this dichotomy, as outlined in the following:

First, dopamine displays much higher affinity for D_3_Rs than for D_2_Rs and thus endogenous dopamine might, at least partly, keeps D_3_Rs occupied even under basal conditions.

Second, animal microdialysis and turnover studies revealed that acute treatment with dopamine agonists such as (-)-pramipexole and (+)-PHNO reduced dopamine turnover, i.e., they decrease extracellular dopamine and increase D3R availability. Administration of antipsychotics (e.g., risperidone, olanzapine, haloperidol, ziprasidone, clozapine, quetiapine), due to antagonism of presynaptic and biosynthesis and release regulating D_2_Rs, leads to several-fold increase of extracellular dopamine. Further, Amato et al. demonstrated that antipsychotics initially suppress dopamine transporter (DAT) activity leading to increase of dopamine in synaptic cleft, a mechanism which represents a further possible alternative way to modulate extracellular dopamine (166). Thus, the increase of extracellular dopamine following antipsychotics with D_2_R antagonism seems to be a likely important factor in the lack or low levels of *in vivo* D_3_R occupancy; given the higher affinity of dopamine for D_3_R vs. D_2_R. Thus, D_2_R antagonist antipsychotics inhibit their own binding at D_3_Rs by increasing extracellular dopamine.

Third, beside the effects on the endogenous dopamine levels, the D3R affinity and selectivity appear to be further factors of importance. All three selective D_3_R antagonists (D_3_R vs. D_2_R selectivity ≥100) such as ABT-925, GSK595809 and SB-277011 (with the *in vitro* low nanomolar D_3_R) affinity produced high D_3_R occupancy in animal or human studies, indicating primary importance of selectivity to achieve D_3_R occupancy *in vivo*.

Example of antipsychotics such as the D_3_R/D_2_R partial agonist cariprazine and the D_2_R/D_3_R antagonist blonanserin shows that, in the presence of relatively high affinity for D_2_Rs, subnanomolar affinity for D_3_Rs appears to be necessary for D_3_R occupancy *in vivo*. Further, cariprazine and the F17464 (subnanomolar affinity for D_3_R with 75-fold D_3_R vs. D_2_R), do not increase extracellular dopamine and hence are able to compete for D_3_Rs vs. extracellular dopamine.

The case of D_2_R/D_3_R partial agonist antipsychotics, aripiprazole and brexpiprazole is somewhat controversial. Both demonstrated low nanomolar affinity for D_2_Rs and D_3_Rs (with D_2_R preference) *in vitro*, with negligible effects on extracellular dopamine *in vivo.* However, both produced no or very low occupancy of D_3_Rs for which the likely explanation is the D_2_R preference.

In conclusion, data reviewed and discussed here regarding the current antipsychotics’ *in vitro* D_2_R/D_3_R affinity vs. their brain D_3_R occupancy *in vivo*, indicate that levels of extracellular dopamine (or its change) in different brain regions is a key factor regarding D_3_R occupancy. On the other hand, the compounds’ high (i.e., subnanomolar) D_3_R affinity and/or high D_3_R vs. D_2_R selectivity are also important determining factors to achieve significant D_3_R occupancy in the brain.

## Author Contributions

BKi and BKr drafted the manuscript with several inputs from IL. All authors were participating in the final editing and critical revision of the article and approved the final version to be published.

## Conflict of Interest

BKi, BKr, and IL were employees of Gedeon Richter Plc.

## Publisher’s Note

All claims expressed in this article are solely those of the authors and do not necessarily represent those of their affiliated organizations, or those of the publisher, the editors and the reviewers. Any product that may be evaluated in this article, or claim that may be made by its manufacturer, is not guaranteed or endorsed by the publisher.

## Footnotes

1
https://pdsp.unc.edu/databases/pdsp.php
